# An improved formula for standard hypoxia tolerance time (STT) to evaluate hypoxic tolerance in mice

**DOI:** 10.1186/s40779-018-0180-7

**Published:** 2018-09-30

**Authors:** Gang Xu, Yu-Qi Gao, Yi-Xing Gao, Gang Wu, Jian-Yang Zhang, Wen-Xiang Gao

**Affiliations:** 1 Institute of Medicine and Hygienic Equipment for High Altitude Region, College of High Altitude Military Medicine, Army Medical University (Third Military Medical University), Chongqing, 400038 China; 2 Key Laboratory of High Altitude Medicine, People’s Liberation Army, Chongqing, 400038 China

**Keywords:** Hypoxia, Standard hypoxic tolerance time, Mice model, Body weight

## Abstract

**Background:**

Hypoxia is a primary cause of mountain sickness and a common pathological condition in patients with heart failure, shock, stroke, and chronic obstructive pulmonary disease (COPD). Thus far, little advancement in countering hypoxic damage has been achieved, and one of the main reasons is the absence of an ideal algorithm or calculation method to normalize hypoxia tolerance scores when evaluating an animal model. In this study, we improved a traditional calculation formula for assessment of hypoxia tolerance.

**Methods:**

We used a sealed bottle model in which the oxygen is gradually consumed by a mouse inside. To evaluate the hypoxia tolerance of mice, the survival time (ST) of the mouse is recorded and was used to calculate standard hypoxia tolerance time (STT) and adjusted standard hypoxia tolerance time (ASTT). Mice administered with methazolamide and saline were used as positive and negative controls, respectively.

**Results:**

Since mice were grouped according to either body weight (BW) or bottle volume, we found a strongly negative correlation between STT and BW instead of between STT and bottle volume, suggesting that different BWs could cause false positive or negative errors in the STT results. Furthermore, both false positive and negative errors could be rectified when ASTT was used as the evaluation index. Screening for anti-hypoxic medicines by using mice as the experimental subjects would provide more credible results with the improved ASTT method than with the STT method.

**Conclusion:**

ASTT could be a better index than STT for the evaluation of hypoxia tolerance abilities as it could eliminate the impact of animal BW.

## Background

Hypoxia is an incidental pathophysiological condition caused by pathological changes including heart failure, shock, carbon monoxide poisoning, chronic obstructive pulmonary disease (COPD) and simple anoxia, such as a high-altitude environment. In people who ascend to a high-altitude location rapidly, hypoxia becomes the main cause of acute mountain sickness (AMS) and can lead to severe conditions such as high-altitude pulmonary edema (HAPE) and high-altitude cerebral edema (HACE) [1].

There is limited effective and appropriate methodology for evaluating the hypoxia tolerance of human beings or model animals and the anti-hypoxic effects of drugs or agents [2]. A hypobaric chamber is the most effective method to create a hypoxia model. However, this device is expensive and can only be installed in a professional hypoxia research institution. In 1992, Lu et al. [3] reported a simple model to simulate a hypoxic environment: the sealed bottle experiment. This model lets an animal consume a constant volume of air in a sealed bottle and generate acute anoxia because of the decreasing oxygen concentration. This model is widely used to evaluate the hypoxia tolerance of subjects [4–6]. A formula is used to normalize animal ST into standard hypoxia tolerance time (STT). This model is widely used in China to evaluate the hypoxic tolerance of animals. However, we found a problem in our earlier experiments: the value of STT is strongly influenced by animal body weight (BW) and may lead to false positive or negative results. Thus, we introduced a new variant of the STT formula related to BW in the STT formula to eliminate this influence.

In this study, we used saline to standardize the BW of mice in the experiment group and used non-processed mice as a control. The ST of the mice that were sealed in the bottle was recorded, and two indices were applied to evaluate their hypoxic tolerance. In addition, methazolamide, which is similar to its analog, acetazolamide, and an effective medicine for treating AMS, was used as a positive control [7]. We revised the former formula and calibrated new parameters that consider the effect of BW. The results obtained using our new formula present a better fitting curve than those BW obtained using the standard curve.

## Methods

### Animals

Male Kunming mice, provided by the Center of Experimental Animals of the Army Medical University, were made to fast with free access to drinking water 12 h before the experiment. The BW of the mice on the day of the experiment were between 13.6 g and 25.4 g. All of the animal protocols were approved by the Animal Care and Use Committee of the Army Medical University (AMU), and adequate measures were taken to minimize animal pain and discomfort.

### Measurement of bottle volumes

Bottles with nominal volume sizes of 150 ml and 250 ml were filled with water until the air inside was removed. The bottles were then tightly sealed with their lids and removed from the water. The water inside the bottles was quantified and recorded.

### Hypoxia tolerance experiment

Hypoxia tolerance was evaluated as described previously [8]. Briefly, each mouse was put into a bottle with a nominal volume size of 150 ml or 250 ml, and 5 g or 10 g of soda lime, respectively, was added to absorb the carbon dioxide and water vapor produced by breathing. Then, the lid was tightly sealed until the mouse’s breathing movement stopped. Both medium BW and low BW mice were administered saline (0.1 ml/10 g BW) for one hour before they were sealed in the bottles. Medium BW mice without saline treatment were used as the control. The ST, i.e., the time from when the mouse was sealed in the bottle to its death, was recorded. STT and adjusted standard hypoxia tolerance time (ASTT) were calculated according to formulas (1) and (2), respectively, as follows.

1 $$ \mathrm{STT}=\mathrm{ST}/\left(\mathrm{V}\hbox{-} \mathrm{BW}/0.94\right) $$

2 $$ \mathrm{ASTT}=\mathrm{ST}\times \mathrm{BW}/\left(\mathrm{V}\hbox{-} \mathrm{BW}/0.94\right) $$

STT: Standard hypoxia tolerance time (min/ml), ASTT: Adjusted standard hypoxia tolerance time (min·g/min), ST: Survival time (min), V: Bottle volume (ml), BW: Body weight of mice (g).

### Statistical analysis

We used an independent *t*-test to compare the means of two groups and one-way ANOVA with the Bonferroni post hoc test to compare the means of three groups. We also used Pearson’s correlation analysis and regression analysis to test the correlations between hypoxia tolerance and BW or bottle volume.

## Results

### Impact of BW on hypoxic tolerance expressed with STT or ASTT

**Fig. 1:**
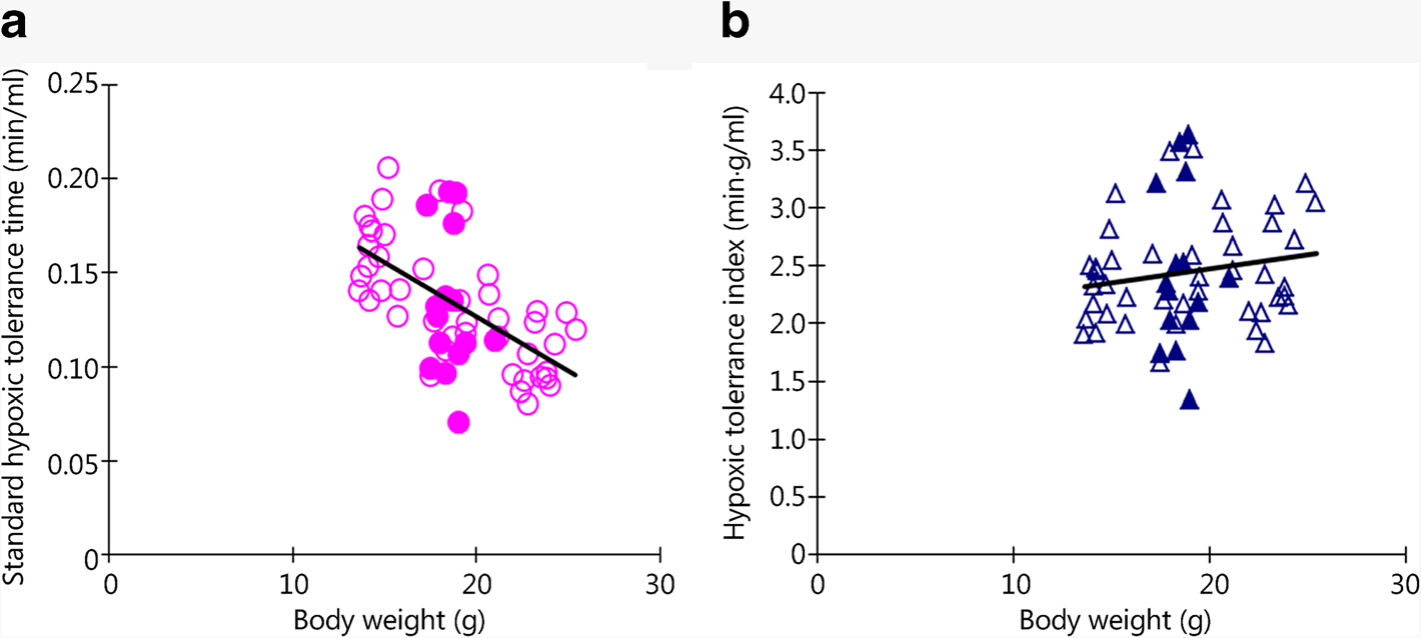
Analysis of the correlation and regression of the hypoxia tolerance indices with the BW of mice. **a**. Hypoxia tolerance was significantly correlated with BW as expressed with STT; **b**. Hypoxic tolerance was not significantly correlated with BW as expressed with ASTT; open marks stand for data obtained with 150 ml bottles, and closed marks stand for data obtained with 250 ml bottles

A strongly negative correlation was found between STT and the BW of mice (*P* = 0.000, regression equation: *y* = − 0.0058*×* + 0.2418, *R*^2^ = 0.3341, Fig. 1a), suggesting that BW differences can significantly impact the experimental results of hypoxia tolerance expressed using STT. To eliminate this impact, we used formula (2) and calculated ASTT as the evaluation index; in contrast to STT, ASTT did not show a significant correlation with the BW of mice (*P* = 0.198, regression equation: *y* = 0.02366*×* + 2.0039, *R*^2^ = 0.0238, Fig. 1b).

We then divided the mice into three groups according to their BW, i.e., low BW group (BW ≤ 16 g), medium BW group (16 g < BW ≤ 21 g) and high BW group (BW ≥ 21 g), and placed them into 150 ml bottles. Concordant with the BWs, which were significantly different among groups (*P* = 0.000, Table 1), the STT was significantly distinct among the groups described above (*P* = 0.000) as well. Using the Bonferroni post hoc test, we further found that the STT was significantly higher in the low group than in either the medium or high group (*P* = 0.000, *P* = 0.000, respectively) and was higher in the medium group than in the high group. These findings confirmed an obvious bias caused by BW on the experiment results expressed with STT. However, no significant difference in ASTT was found among the 3 groups (*P* = 0.392, Table 1), indicating that the effect of BW had been eliminated in formula (2), and ASTT was the more appropriate index for the evaluation of hypoxic tolerance.

**Table 1:** Impact of BW on the hypoxic tolerance indices of mice ($\overline{x}$±*s*)

Group	BW (g)	Bottle volume (ml)	ST (min)	STT (min/ml)	ASTT (min·g/ml)
Low BW (*n* = 15)	14.55 ± 0.68	158.1 ± 2.7	22.84 ± 3.09	0.1602 ± 0.0222	2.33 ± 0.343
Medium BW (*n* = 13)	19.00 ± 1.29^△^	158.1 ± 2.7	18.66 ± 4.18^△^	0.1349 ± 0.0286^△^	2.562 ± 0.551
High BW(*n* = 15)	23.33 ± 1.10^△▲^	158.1 ± 2.7	14.02 ± 2.19^△▲^	0.1052 ± 0.0168^△▲^	2.46 ± 0.435

ST. Survival time; STT. Standard hypoxia tolerance time; ASTT. Adjusted standard hypoxia tolerance time; BW. Body weight. △*P* < 0.01 compared with low group; ▲ *P* < 0.01 compared with medium group

### Impact of bottle volume on hypoxic tolerance expressed with STT or ASTT

**Fig. 2:**
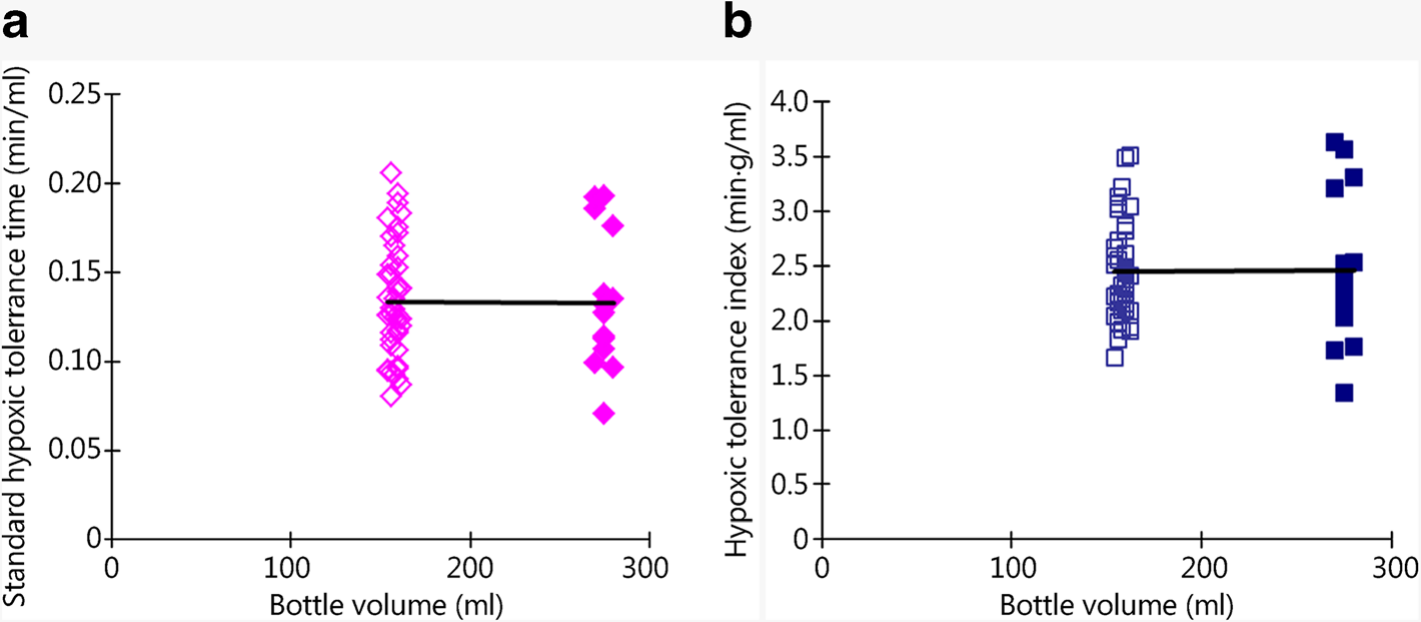
Analysis of the correlation and regression of the hypoxic tolerance indices with bottle volume. Both indices, **a**. STT and **b**. ASTT, showed no significant correlation with bottle volume; open marks stand for data obtained with 150 ml bottles, and closed marks stand for data obtained with 250 ml bottles

In contrast to BW, bottle volume was not significantly correlated with either STT (*P* = 0.267, Fig. 2a) or ASTT (*P* = 0.104, Fig. 2b), showing that bottle volume did not affect either STT or ASTT. Furthermore, we compared the mice treated in 150 ml and 250 ml bottles and did not find significant differences in hypoxia tolerance expressed with either STT (*P* = 0.314, Table 2) or ASTT (*P* = 0.443, Table 2), suggesting that hypoxic tolerance was not correlated with air volume and was most likely associated with the characteristics of the mice.

**Table 2:** Impact of bottle volume on hypoxic tolerance indices in mice ($\overline{x}$±*s*)

Group	BW (g)	Bottle volume (ml)	ST (min)	STT (min/ml)	ASTT (min·g/ml)
150 ml group (*n* = 13)	19.00 ± 1.29	158.1 ± 2.7	18.66 ± 4.18	0.13 ± 0.029	2.56 ± 0.551
250 ml group (*n* = 15)	18.56 ± 0.90	275 ± 3.3	33.87 ± 9.54	0.13 ± 0.038	2.46 ± 0.689

STT. Standard hypoxia tolerance time; ASTT. Adjusted standard hypoxia tolerance time

### Eliminate false positive results with formula (2)

As BW was found to have a significant impact on hypoxia tolerance expressed with STT, we inferred that the BW of animals in this experiment could lead to systemic errors, i.e., if mice in the treatment group had lower BWs than those in the control group, we could obtain a false positive result, whereas if mice in the treatment group had higher BWs than those in the control group, we could obtain a false negative result.

First, we performed experiments to observe whether lower BWs could cause false positive results and whether these false positive results could be eliminated with formula (2). As the BW was matched between the control group and saline group 1 (medium BW), there was no significant difference in STT between the two groups, showing that saline did not affect hypoxia tolerance in the mice. However, as the BWs of mice were significantly lower in saline 2 group (low BW), the STT was significantly higher in this group than in the control group (*P* = 0.000, Table 3), which was obviously a false positive result. This finding suggests that lower BW can bring about a false positive result when using STT as the evaluation index. As expected, the ASTT was not significantly different between the three groups (*P* = 0.781, Table 3), confirming that the false positive findings caused by lower BW could be avoided by using ASTT.

**Table 3:** Effects of BW on hypoxic tolerance indices in mice ($\overline{x}$±*s*)

Group	BW (g)	Bottle volume (ml)	ST (min)	STT (min/ml)	ASTT (min·g/ml)
Control (*n* = 12)	18.66 ± 0.70	158.2 ± 2.9	15.67 ± 2.03	0.11 ± 0.0147	2.12 ± 0.297
Medium BW (Saline 1 (*n* = 12)	18.57 ± 0.49	158.2 ± 2.9	16.08 ± 5.05	0.12 ± 0.0369	2.15 ± 0.636
Low BW (Saline 2(*n* = 14)	14.40 ± 0.52*	158 ± 2.7	22.14 ± 2.77	0.16 ± 0.0189*^△^	2.23 ± 0.255

ST. Survival time; STT. Standard hypoxia tolerance time; ASTT. Adjusted standard hypoxia tolerance time; BW. Body weight. *P < 0.01 compared with saline group; △ *P* < 0.01 compared with medium BW group (saline 1 group)

### Eliminate false negative results with formula (2)

Methazolamide is an analog of acetazolamide and has been proven to be effective in preventing the symptoms of AMS in an experiment with human beings [9]. Methazolamide is also neuroprotective in ischemic injured mice by inhibiting the release of apoptotic factors [10, 11]. Therefore, mice administered with methazolamide were used as the positive control in this test. To test the false negative results caused by higher BW, mice were administered 100 mg/kg of methazolamide one hour before they were sealed in the bottles. Medium BW mice were administered saline (0.1 ml/10 g BW) as the control. According to previous reports, when the BWs of mice were in the medium range, the STT was significantly higher in methazolamide 1 group (medium BW) than in the saline group, confirming that methazolamide has a significant anti-hypoxic effect in mice. However, the STT was not significantly different between methazolamide 2 group (high BW) and the saline-treated group (*P* = 0.396), as the BW in methazolamide 2 group were significantly higher than those in the saline group (*P* = 0.005), suggesting that false negative results can be caused by higher BW. Then, we used formula (2) to calculate ASTT and found no significant difference between methazolamide 1 (medium BW) and methazolamide 2 (high BW) group (*P* = 1.000, Table 4), whereas both groups had significantly higher ASTT values than the saline group (*P* = 0.000, *P* = 0.000, respectively, Table 4).

**Table 4:** Effects of methazolamide and BW on hypoxic tolerance indices in mice ($\overline{x}$±*s*)

Group	BW (g)	Bottle volume (ml)	ST (min)	STT (min/ml)	ASTT (min·g/ml)
Saline (*n* = 13)	18.31 ± 0.60	158 ± 2.8	15.12 ± 2.06	0.11 ± 0.0146	2.00 ± 0.283
Methazolamide 1 (Medium BW) (*n* = 13)	18.68 ± 0.68	158 ± 2.8	20.62 ± 3.17	0.15 ± 0.0218*	2.78 ± 0.367*
Methazolamide 2 (High BW) (*n* = 13)	22.59 ± 1.03*	158 ± 2.8	16.22 ± 3.29	0.12 ± 0.0241^△^	2.73 ± 0.457*

ST. Survival time; STT. Standard hypoxia tolerance time; ASTT. Adjusted standard hypoxia tolerance time; BW. Body weight. * *P* < 0.01 compared with saline group; △ *P* < 0.01 compared with methazolamide group 1 (medium BW)

## Discussion

In this study, we applied a simple method to evaluate the hypoxia tolerance of mice and investigated the influence of BW and bottle volume on hypoxia tolerance indices. To eliminate the interference of BW and bottle volume with this model, we developed a modified formula (formula (2)) to calculate an adjusted hypoxia tolerance time.

Using the methazolamide treated positive control and saline treated negative control, we were able to evaluate the hypoxia tolerance of mice with this simple method. The hypoxia tolerance of animals has been evaluated with STT, i.e. the number of minutes that one unit volume of air could support animal survival, in previous studies [6, 8]. Therefore, according to the formula, STT should be determined not by animal ST but by bottle volume (V) and BW of the animal. In other words, bottle volume and BW are the primary factors that affect this hypoxia tolerance experiment. In this study, we found that the impact of BW and bottle volume on the results of this experiment expressed with STT were different: (1) STT was negatively correlated with animal BW, and (2) bottle volume did not impact STT.

Bottles with nominal volumes of 150 ml and 250 ml were used as the experimental vessels in our study, and the actual volumes could have been slightly different among all of the bottles. For example, the actual volumes of the 150 mL-sized bottles used in this study ranged between 154 ml to 162 ml. As the animals’ BWs and volumes were different, the air remaining in the bottles for the animals to breathe would accordingly be different. However, bottle volume was not significantly correlated with either STT or ASTT in this study, which implied that a slight deviation in bottle volumes did not affect the experimental results.

Excluding pathological states such as obesity, BW is usually a critical factor in animal experiments. In formula (1), animal volume, expressed as the mouse’s BW over its average density (0.94), was subtracted from the bottle volume to eliminate the impact of the air volume displaced by the animal’s body inside the bottle. In fact, BW has a stronger effect than animal volume, as shown by STT. Our study suggests that animals of different BWs showed different hypoxia tolerance abilities, which was in accordance with previous reports. For example, hypoxic-ischemic encephalopathy occurs in up to 60% of low birth weight infants [12, 13], which is related to the immaturity of brain blood vessels and the dysfunction of brain blood regulation, as well as cellular oxidation-reduction in newborns with low BW [14, 15]. Moreover, obese humans are more likely to suffer from AMS after ascending to high altitudes [16, 17]. The mechanisms involved in the association between hypoxic tolerance and BW in adult animals, which may be related to blood oxygenation [16], energy turnover rate, and lipid-glucose metabolism [18], are not clear at present. In pharmacodynamic studies, chemical compounds are administered to animals for 7 days or longer. During the administration period, the BWs of animals could deviate gradually and lead to errors because of the inter-group or inner-group BW distribution of animals. As a result, false negative or positive findings could occur. Therefore, the BWs of animals in the treatment group and the control group could affect the results after the administration process, and the experiment may fail. To eliminate the impact of BW on experiment results, we introduced another index, ASTT, calculated with formula (2). ASTT is defined as the number of minutes that a unit volume of air can support the survival of one unit weight of an animal’s body. As ASTT was used to evaluate the hypoxia tolerant abilities of mice in sealed bottles, it was not significantly different between groups with different BWs, showing that this formula can successfully eliminate the impact of BW on the experimental results and avoid false positive or negative findings. To verify that this formula was effective in eliminating false negative and positive results caused by different BWs, we intraperitoneally injected saline and 100 mg/kg methazolamide immediately before the hypoxic treatment of mice with different BWs. Our results confirmed that when there were different BWs between groups, false negative or positive findings caused by STT could be avoided with ASTT.

## Conclusions

In summary, this experiment is a simple method to evaluate the hypoxia tolerance abilities of mice. This study can be used as a preliminarily screening for anti-hypoxic medicines before mechanistic research and studies with human subjects. When the BWs in all of the experimental groups are closely matched, STT can be used as an index of hypoxia tolerance. Otherwise, ASTT could be a more appropriate index for the evaluation of hypoxia tolerance, as it could eliminate the impact of BW.

## Abbreviations

AMS: Acute mountain sickness
AMU: Army medical university
ASTT: Adjusted standard hypoxia tolerance time
BW: Body weight
COPD: Chronic obstructive pulmonary disease
HACE: High-altitude cerebral edema
HAPE: High-altitude pulmonary edema
ST: Survival time
STT: Standard hypoxia tolerance time
